# Spontaneous Adverse Drug Reaction Reporting of Congenital Malformations: A Danish National Register Study

**DOI:** 10.3390/ph18060917

**Published:** 2025-06-18

**Authors:** Ulrik Lausten-Thomsen, Rasmus Huan Olsen, Michael Christiansen, Paula L. Hedley, Ida Marie Heerfordt, Jon Trærup Andersen, Christina Gade

**Affiliations:** 1 Department of Neonatology, Copenhagen University Hospital—Rigshospitalet, 2100 Copenhagen, Denmark; 2 Department for Congenital Disorders, Statens Serum Institut, 2300 Copenhagen, Denmark; 3 Department of Clinical Pharmacology, Copenhagen University Hospital—Bispebjerg and Frederiksberg, 2400 Copenhagen, Denmark; 4 Department of Clinical Medicine, University of Copenhagen, 2200 Copenhagen, Denmark; 5 Department of Biomedical Science, University of Copenhagen, 2200 Copenhagen, Denmark; 6 Department of Epidemiology, School of Public Health, University of Iowa, Iowa City, IA 52242, USA

**Keywords:** adverse drug reaction reporting systems, congenital abnormalities, drug-related side effects and adverse reactions, infant, newborn, pharmacovigilance

## Abstract

**Background/Objectives**: Maternal use of medication during pregnancy may have teratogenic effects, as seen with drugs like thalidomide, valproate, and phenytoin. Despite rigorous testing, both new and established drugs still pose a risk of teratogenesis, particularly if the teratogenic effects are probabilistic and not deterministic. Public health organizations maintain registers to centralize and evaluate adverse drug reactions (ADR). However, underreporting in these registries can obscure the signals of drug-related congenital malformations. This study aims to evaluate potential ADR-associated congenital malformations in Denmark over the past decade; **Methods**: An observational cross-sectional study was conducted using data from the national Danish Medicines Agency’s pharmacovigilance database, which includes all spontaneous ADR reports submitted to the Danish Medicines Agency from 1 July 2013 to 30 June 2023. Maternal antenatal drug use was identified, and reported ADRs were assessed for congenital malformations; **Results**: We identified reports of potential ADR-related congenital malformations in 75 children, with 92 diagnoses as classified by ICD-10. Eighty-five different drugs from 58 ATC codes were implicated. Only three diagnoses were reported in five or more children. The reports were generally sporadic, with no new signals detected; **Conclusions**: Public awareness is crucial when novel threats arise from medications, infections, or technologies, as these may pose risks to unborn children. Ongoing monitoring of potential ADR-related congenital malformations remains a critical component of public health. Given the potential underreporting, we encourage a low threshold for reporting ADRs based on suspicion alone, with final causality assessments made by health authorities.

## 1. Introduction

Embryofoetal development is a highly complex process in which any disruption from external influences may have detrimental effects. Among several recognised risk factors, maternal medication use during pregnancy represents a well-known potential risk factor for congenital malformations due to teratogenic effects [1].

A classic example of drug-induced congenital malformations is the thalidomide tragedy of the late 1950s and early 1960s. Thalidomide was initially marketed as a sedative and treatment for morning sickness in pregnant women. However, it became evident that thalidomide exposure during pregnancy caused a range of severe congenital malformations, collectively known as thalidomide embryopathy [2,3]. Several other examples of otherwise useful drugs with potentially devastating teratogenic side effects include warfarin (anticoagulant) [4], valproate and phenytoin (antiepileptic) [5,6], and isotretinoin (retinoid) [7].

In particularly, the thalidomide tragedy led to significant changes in drug regulation worldwide, including requirements for stricter testing for teratogenic effects before a drug could obtain a marketing authorization. However, both new and old well-known drugs may still carry a risk of teratogenic side effects that may not have been anticipated or discovered during pre-market testing; especially if the malformation is rare and the effect caused by the drug is not deterministic but probabilistic.

Accordingly, there is a continuous need for pharmacovigilance even after a drug has obtained marketing authorization. Most national public healthcare organizations own registers designed to centralize and evaluate adverse drug reactions (ADRs), just as pharmaceutical manufacturers are required to monitor their drugs and report these drug monitoring results to health authorities. Especially when screening for rare and sporadic malformations, central registers are a prerequisite for signal generation that may otherwise be lost due to the scarcity of the event. Unfortunately, many side effect registers tend to suffer from overall underreporting, especially when it comes to side effect reports for the paediatric population [8], potentially leading to loss of signals in surveillance of pregnancy-drug-related congenital malformations.

The aim of this study was to evaluate the number and categories of reported potential ADR-associated congenital malformation reported in Denmark over the last decade. Furthermore, we evaluated the potential usefulness of the Danish Medicines Agency’s pharmacovigilance database for the signal generation of ADR-related congenital malformations. Finally, we aimed to identify new signals for potential ADR-related congenital malformations.

## 2. Results

### 2.1. Overall Findings

We identified reports of potential ADR-related teratogenic congenital malformations in 75 individual children under the age of two years: 45 boys (60%; 95% CI: 44–81), 29 girls (39%; 95% CI: 26–56), and 1 with missing information on sex. No significant difference in the gender distribution was observed (*p* = 0.09). The 75 identified children combined for a total of 63 diagnoses, classifiable as per ICD-10. All serious adverse drug reactions were considered as serious, and 8 of the 75 included children were reported to have died.

### 2.2. Data Quality

A total of 228 reports were identified on 75 individuals, as each case could have multiple reports, e.g., in the case of several suspected drugs, or the same case could be reported several times by healthcare professionals, laymen, and lawyers. Each child has a unique, individual Danish social security number which was used on the original ADR report, which allowed for identification of duplicated cases, and which was reported several times. The median number of reports was 1 (IQR 1–4; range 1–24). Of the 75 cases, 12 (16%) did not have a report completed by a physician or other healthcare professional.

In all cases, an ICD-10 classification of suspected congenital malformations could be made, and similarly in all cases, a corresponding ATC code for the suspected drug could be identified. In 5 reports (2.1%), drug indications were not reported. In all cases, full data on (i) date of report, (ii) age of patient, (iii) suspected drug, and (iv) suspected congenital malformations were available. There were no available data on maternal age and/or diagnoses. Due to the anonymized nature of the dataset obtainable through the Danish Medicines Agency’s pharmacovigilance, linkage to other Danish health registries was not possible.

### 2.3. Suspected Teratogenic Effects

Only three diagnoses/malformations were reported in five or more children, i.e., eight cases of ventricular septal defect (ICD-10: DQ210), eight cases of hypospadias (ICD-10: DQ549), and five cases of congenital hand malformation (ICD-10: DQ681). For the 8 cases of ventricular septal defect, 11 different drugs were reported in total, and no drugs were reported in more than one case. For the 8 cases of hypospadias, 8 different drugs were reported, and no drug was reported in more than one case. For the 5 cases of congenital hand malformation, 13 different drugs were reported, and once again, no drug was reported more than once. Therefore, no clear evidence of teratogenic effects was found. Given the scarcity of the more seldomly occurring malformations (i.e., <5) and the relatively high number of reported drugs, no additional signals were observed for any other malformations and/or drugs.

### 2.4. Observed Malformations and Reported Drugs

A list of the various reported congenital malformations aggregated by organ system and/or ICD-10 code, together with the responding number of drugs potentially responsible for ADR-associated congenital malformation, is provided below (Table 1).

A total of 85 individual drugs were reported as possibly linked to the malformations (Table 1), but when classifying the drugs per ATC group, this number decreased to 58. To provide greater detail on the active substances, Table 2 presents the complete list of all individual active substances suspected to be associated with congenital malformations, including their ATC codes and number of different drugs within the ATC code. In total, 85 different drugs from 58 unique ATC codes were reported to be potentially linked to congenital malformations.

## 3. Discussion

In this national, decade-long observational cohort study, we identified 92 congenital malformations associated with adverse drug reactions in 75 children. This corresponds to 75 children out of approximately 600,000 births during the study period, or 0.013%. By contrast, the overall prevalence of congenital malformations in Denmark is approximately 3.5% [9,10], which aligns with other European estimates [11,12]. This indicates that only approximately 0.4% of congenital malformations are linked to the ADR report. During the same period, prescription medication was used in in approximately 65% of Danish pregnancies [13], meaning that a large proportion of pregnancies resulting in congenital malformations were likely exposed to prenatal medication. The low reporting rate highlights either that most prenatal medications are not associated with congenital malformations, or that there is substantial underreporting or limited sensitivity in identifying and attributing suspected ADRs in this population. Notably, the ratio of ADR-associated congenital malformations has been estimated to be approximately 0.2% [14], which is close to our observed rate and may reflect both underreporting and the inherent difficulty of establishing causal links between prenatal drug exposure and congenital outcomes. Importantly, due to the register’s design and the 100% coverage of national, unique social security numbers for personal identification in Denmark, we were able to obtain the age for 100% of the ADR reports. This greatly enhances the use of the ADR register from a paediatric/neonatology perspective, when compared with other systems/registers that for many reports do not have the possibility of age estimation, e.g., the FDA Adverse Event Reporting System [8]. Together with 100% coverage in potential reaction (i.e., the malformation) and drug use, the register appears to be sufficiently well constructed to capture potential ADR-associated malformations.

Our findings of more boys than girls were not significant, and this observation is likely to represent a simple variant, but it may also reflect that boys have an overall higher risk of congenital malformations [15,16]. Nor is it surprising that hypospadias, ventricular septal defect, and hand/finger malformations were the most commonly reported in our study, as these malformations are the most commonly reported in the general population [9].

We did not suspect any new signals with regards to ADR-associated congenital malformations. Although each individual report may represent the true ADR-induced congenital malformation, we did not find any statistically significant signals. However, in this study we were not able to assess whether the reports contributed to signals when combined with international databases such as the EMA Eudravigilance system or the WHO VigiBase. Given that the Danish reports are integrated within the EMA EudraVigilance system, future research may provide further elucidation on this matter; however, this falls beyond the scope of the current study. Importantly, we did find some reports that seem very unlikely to represent true ADR-induced congenital malformation; such malformations were reported to be caused by glucose or sodium chloride solution.

A strength of this study is the use of a nationwide, register-based methodology that captures comprehensive real-world clinical data, minimizing ascertainment bias. The data have nationwide coverage and a high degree of data completion, thereby allowing a comprehensive national evaluation of the epidemiological characteristics of potential ADR-associated congenital malformations in Denmark. On the other hand, the lack of access to maternal health data is a limitation in the current methodology; having full access to information on e.g., maternal age and full medical history, may have provided hints to ADR–congenital malformation causality that the current Danish ADR register does not capture.

However, this study is based on data from a system that per design captures only spontaneously reported ADRs, and therefore inherently represents a risk of underreporting. Conversely, overreporting of particularly congenital malformations may also occur, as both laymen and healthcare professionals may be psychologically prone to look for an explanation in the traumatic situation of an unexpected congenital malformation. Conceptually, the data can only demonstrate possible associations but cannot definitively establish a direct causal link between the medications and the adverse events, i.e., the malformation.

Furthermore, congenital malformations are fortunately rare, and observational data on rare events do require large sample sizes to adequately demonstrate an association between maternal drug use and subsequent malformation. Assuming a frequent malformation of 1% (e.g., male hypospadias) and a birth cohort of 60,000 children per year (as is the case in Denmark), the ‘naturally occurring’ number of hypospadias would be approximately 300/year. The introduction of a new teratogenic drug should then cause an additional approximately 50 cases of hypospadias for the increase to be significant (χ^2^ test, *p* < 0.05), and thereby needs to be taken by at least 100 pregnant women or approximately 0.17% of pregnancies.

If the causality between maternal use of this hypothetical drug and subsequent creation of the malformation is not deterministic, but rather probabilistic, and the drug use only increases the risk of the malformation with e.g., 20%, the drug needs to be taken by 500 women or in 0.8% of pregnancies. It is therefore perfectly possible that the potential teratogenic effect of an uncommonly used drug could remain undetected in smaller birth cohorts.

Birth defects have always been a feared outcome of pregnancy, but historically, maternal and early neonatal demise from various other causes, such as infection, famine, and premature birth, were more common. With increasing affluence, maternal and neonatal deaths from infectious diseases have decreased, making birth defects a proportionally more significant cause of infant mortality. It is now well known that maternal drug use may increase the risk of congenital malformations, and the rise in technologies for assisted reproduction also appears to increase the risk of birth defects. Therefore, surveillance of ADR-associated congenital malformations is crucial.

Moreover, effective surveillance of birth defects can alleviate public anxiety when new threats arise, whether from new medications, infections, or novel technologies. The public has a legitimate need to know if these new factors pose risks to themselves or their unborn children. Without comprehensive registries, healthcare professionals face these inquiries without adequate information, which only heightens public fears. Therefore, the continuous surveillance of potential ADR-associated congenital malformations remains an important aspect of public health.

Some ADR databases are better for certain types of signal generation than others [17]. The Danish Medicines Agency’s pharmacovigilance database appears to represent a useful register in relation to signal generation with a high level of detail coverage, particularly regarding the exact age, drug use, and potential malformation. The disadvantage is that the register only covers a small population and therefore makes only a small contribution from an international perspective. Nonetheless, due to the rarity of most malformations, strengthened international collaboration between national registers may enhance detection ability in cases of uncommonly used drugs and rare malformation events. These should include cooperation to improve the quality of reports, for example, introducing a hard stop if the age and route of drug administration are not stated when reporting ADRs [8]. In addition, given the risk of underestimation, we encourage a low threshold for reporting ADRs based on suspicion alone, while the final assessment of causality should take place on the part of the health authorities.

## 4. Materials and Methods

### 4.1. Study Sample

We conducted an observational cross-sectional study by collecting data from the Danish Medicines Agency’s pharmacovigilance database, which contains all spontaneous ADR reports submitted to the Danish Medicines Agency from 1 July 2013 to 30 June 2023.

### 4.2. Case Classification

The Danish Medicines Agency characterizes an ADR as ‘an unwanted reaction of a medicine’ [18]. An ADR indicates a potential association between the medication and the observed adverse outcome, with reports being based on ‘suspected’ rather than ‘proven’ causal relationships [18].

As defined by the European Medical Agency [19], reported incidents were deemed serious if they resulted in death, were life-threatening, required hospitalization or an extension of existing hospitalization, led to persistent or significant disability or incapacity as determined by the reporter, or were associated with a congenital anomaly or birth defect. Suspected ADRs not meeting these criteria were classified as non-serious. The Danish ADR database classifies five groups of authorized reporters: physicians (including GPs, MDs, and dentists), pharmacists (working in community or hospital settings), other healthcare professionals (such as nurses, pharmaceutical company employees, and healthcare assistants), lawyers (including those from patient injury insurers and law firms), and consumers or other non-healthcare professionals (such as patients, relatives, and the general public).

We ascertained all ADRs reported on children aged 0–2 years, thereby assuming that all congenital malformations were detected before the age of 2 years. For each case, we identified drug trade names, ADRs categorized using the Medical Dictionary for Regulatory Activities terminology at the System Organ Class (SOC) level [20], the severity of the reactions, infant age, infant sex, information about the reporter, and the date of ADR report submission.

We included cases if the ADR report was within the following categories: (1) drugs that were registered explicitly as given prenatally, (2) registered as leading to foetal effect, (3) registered as leading to a clearly congenital condition, or (4) registered as being given for an explicit prenatal/maternal reason. See also the Appendix A for a full list. The reported ADRs were ascertained as being a congenital malformation if the corresponding ICD-10 code was within the ICD-10 interval of Q00–Q999 (Congenital malformations, deformations, and chromosomal abnormalities) or represented syndromes or genetic aberrations classified elsewhere in the ICD-10 classification system.

For each drug trade name, the corresponding Anatomical Therapeutic Chemical (ATC) classification code per WHO was added using the Danish Medicines Catalogue [21,22].

### 4.3. Setting and Relevant Context

The study was conducted within the socio-cultural and healthcare framework of Denmark, a nation characterized by a comprehensive and free welfare system. In Denmark, healthcare services, including those for expecting mothers and infants, are accessible to all. Furthermore, the Danish Health Registers are widely regarded as exceptionally reliable, consistently covering all demographics without disparities in gender, social group, or geography [23].

### 4.4. Statistics

Statistical analyses were performed using R (version 4.3.0 “Already Tomorrow”). Prevalence rate ratios (PRRs) and 95% confidence intervals (CIs) were estimated using Poisson regression.

### 4.5. Ethics

The anonymized dataset from the national Danish ADR database was provided by the Danish Medicines Agency in accordance with the Public Access Act, Section 11, Subsection 1 [24]. As the study used data from administrative registers, ethical approval and individual informed consent were not required as per Danish regulations for register-based studies. For privacy reasons, results for groups with fewer than five individuals are not reported.

## Figures and Tables

**Table 1:** Various reported congenital malformations aggregated by organ system and/or ICD-10 code.

Congenital Malformation	Different Diagnoses	Reported Patients *	Reported Drugs ^#^	Reported Drugs as per ATC ^§^
CNS (DQ00-DQ079):	4	6	7	5
Eyes, ears, face, and neck (DQ10-DQ189)	8	7	20	18
Cardiao-vascular (DQ21-DQ289)	9	19	20	18
Respiratory (DQ30-DQ349)	1	1	3	3
Oro-facial (DQ35-DQ379)	2	2	2	2
Gastrointestinal (DQ38-DQ459)	6	8	8	7
Genital (DQ50-DQ569)	4	13	19	15
Uro-renal (DQ60-DQ649)	3	4	8	8
Bones, muscles, and tissues (DQ65-DQ799)	20	26	37	29
Other malformations (DQ80-DQ899)	2	2	2	2
Chromosomal (DQ90-DQ999)	1	1	2	1
Syndromes classified elsewhere	3	3	3	3
Total number	63	75	85	58

* a patient can a several MCA and consequently the categories can sum up to more than 100%; # a drug can be suspected in several cases in the same way that several drugs can be suspected in a unique case; § idem.

**Table 2:** List of drugs, and the corresponding ATC code, reported to potentially be associated with congenital malformation.

ATC Code	Active Substance	N^o^ Drugs *
A02BB01	Misoprostol	1
A04AA01	Ondansetron	4
A10BJ02	Liraglutide	1
C03CA01	Enalapril	1
C10AA01	Simvastatin	5
D07AB02	Hydrocortisone butyrate	1
D07AC01	Betamethasone	1
G02BA03	Levonorgestrel	1
G03AA09	Ethinylestradiol and norgestimate	1
G03AA12	Ethinylestradiol and gestodene	2
H02AB02	Budesonide	1
H02AB04	Methylprednisolone	7
H03AA01	Levothyroxine	4
J07BM01	Human papillomavirus vaccine	2
L01AA01	Cyclophosphamide	1
L01BA01	Methotrexate	1
L01CA02	Vincristine	1
L01DB01	Doxorubicin	1
L01DB03	Epirubicin	1
L01XX02	Asparaginase	1
L04AB01	Etanercept	3
L04AB02	Infliximab	3
L04AB05	Certolizumab pegol	1
L04AA27	Fingolimod	1
L04AC16	Ustekinumab	1
L04AX01	Azathioprine	1
M05BA03	Pamidronic acid	1
M09AX07	Nusinersen	1
N02AA05	Oxycodone	1
N02BE01	Paracetamol (Acetaminophen)	4
N02CC01	Sumatriptan	1
N03AB02	Phenytoin	6
N03AG01	Valproic acid	3
N03AX09	Lamotrigine	5
N03AX14	Levetiracetam	3
N03AX16	Pregabalin	1
N05AF05	Zuclopenthixol	3
N05AH04	Quetiapine	9
N05AN01	Lithium	2
N05AX12	Aripiprazole	1
N05BA04	Oxazepam	4
N05CF01	Zopiclone	4
N06AA10	Nortriptyline	8
N06AB03	Fluoxetine	2
N06AB04	Citalopram	3
N06AB05	Paroxetine	3
N06AB06	Sertraline	9
N06AB10	Escitalopram	1
N06AX11	Mirtazapine	1
N06AX16	Venlafaxine	5
N06AX21	Duloxetine	2
N06BA09	Atomoxetine	1
P01BA02	Hydroxychloroquine	2
P01BB51	Proguanil and atovaquone	1
R06AD02	Promethazine	1
R06AE07	Cetirizine	1
V03AF07	Glucose	1
V04CB01	Sodium chloride	1

ATC: Anatomical Therapeutic Chemical code; * number of drugs with the same ATC code.

## Data Availability

The datasets generated and analysed during the current study are not publicly available due to Danish GDPR but are available from the corresponding author on reasonable request.

## Appendix A

The following parameters were used for extracting data from the Danish ADR database:

- Registered explicitly as given prenatally (10: drug exposure in utero, drug exposure via mother, exposure during pregnancy, foetal exposure during delivery, foetal exposure during pregnancy, foetal exposure timing unspecified, maternal drugs affecting foetus, maternal drug exposure, maternal exposure during pregnancy, maternal therapy to enhance foetal lung maturity);
- Registered as leading to foetal effect (20: abortion induced, Apgar score abnormal, Apgar score low, asphyxia, foetal anticonvulsant syndrome, foetal chromosome abnormality, foetal disorder, foetal distress syndrome, foetal growth restriction, foetal heart rate abnormal, foetal heart rate deceleration abnormality, foetal heart rate decreased, foetal heart rate disorder, foetal hypokinesia, foetal monitoring abnormal, low birth weight baby, premature baby, premature delivery, premature labour, small for dates baby);
- Registered as leading to a clearly congenital condition (71: congenital, familial and genetic disorders, anal atresia, anomaly of external ear congenital, atrial septal defect, atrioventricular septal defect, brachydactyly, cardiac septal defect, cleft lip and palate, cleft palate, coloboma, congenital anomaly of adrenal gland, congenital aortic anomaly, congenital brain damage, congenital diaphragmatic hernia, congenital eyelid malformation, congenital foot malformation, congenital gastric anomaly, congenital genital malformation male, congenital hand malformation, congenital heart valve disorder, congenital hydronephrosis, congenital inguinal hernia, congenital jaw malformation, congenital megacolon, congenital musculoskeletal disorder, congenital oesophageal anomaly, congenital spinal fusion, craniofacial deformity, craniosynostosis, cryptorchism, deformity thorax, digeorge’s syndrome, duodenal atresia, dysmorphism, ear malformation, ebstein’s anomaly, epispadias, external auditory canal atresia, foetal chromosome abnormality, heart disease congenital, hemivertebra, holoprosencephaly, hypertelorism, hypospadias, limb reduction defect, low set ears, meningocele, meningomyelocele, micropenis, microtia, mitral valve incompetence, oesophageal atresia, osteochondrodysplasia, otospondylomegaepiphyseal dysplasia, pectus excavatum, phalangeal hypoplasia, poland’s syndrome, polydactyly, pulmonary hypoplasia, pulmonary valve stenosis congenital, renal fusion anomaly, retrognathia, spina bifida, spinal muscular atrophy, syndactyly, talipes, telangiectasia, umbilical cord abnormality, vacterl syndrome, ventricular septal defect);
- Registered as being given for an explicit prenatal/maternal reason (9: contraception, induced labor, labour induced, labor induction, labour pain, labour stimulation, pre-eclampsia, pregnancy induced hypertension, uterine contractions during pregnancy).
